# Smart and Biodegradable Polymers in Tissue Engineering and Interventional Devices: A Brief Review

**DOI:** 10.3390/polym17141976

**Published:** 2025-07-18

**Authors:** Rashid Dallaev

**Affiliations:** Department of Physics, Faculty of Electrical Engineering and Communication, Brno University of Technology, Technická 2848/8, 61600 Brno, Czech Republic; rashid.dallaev@vut.cz

**Keywords:** smart biomaterials, biodegradable polymers, regenerative medicine, shape-memory polymers (SMPs), controlled degradation, tissue engineering scaffolds, drug delivery systems, polymeric occluders

## Abstract

Recent advancements in polymer science have catalyzed a transformative shift in biomedical engineering, particularly through the development of biodegradable and smart polymers. This review explores the evolution, functionality, and application of these materials in areas such as tissue scaffolding, cardiovascular occluders, and controlled drug delivery systems. Emphasis is placed on shape-memory polymers (SMPs), conductive polymers, and polymer-based composites that combine tunable degradation, mechanical strength, and bioactivity. The synergy between natural and synthetic polymers—augmented by nanotechnology and additive manufacturing—enables the creation of intelligent scaffolds and implantable devices tailored for specific clinical needs. Key fabrication methods, including electrospinning, freeze-drying, and emulsion-based techniques, are discussed in relation to pore structure and functionalization strategies. Finally, the review highlights emerging trends, including ionic doping, 3D printing, and multifunctional nanocarriers, outlining their roles in the future of regenerative medicine and personalized therapeutics.

## 1. Introduction

Smart polymers with tunable mechanical properties, precise geometric control, and environmental sensitivity are promising candidates for scaffolds and stents in cell therapy. These materials can serve as carriers for cells, drugs, or proteins, enabling stimulus-triggered release. Additive manufacturing (AM) offers a pathway to bridge the gap between biomaterial innovation and clinical cell therapy applications [1]. By tailoring the properties of smart materials, it is possible to modulate stem cell differentiation, supporting tissue regeneration and personalized medicine. For instance, smart scaffolds can facilitate targeted differentiation of stem cells into desired tissue types [2]. Self-healing smart materials are also being explored for use in bioprinting, potentially influencing induced pluripotent stem cell (iPSC) differentiation and providing minimally invasive solutions for tissue repair and regeneration [3,4].

The convergence of smart and biodegradable polymers has transformed the field of regenerative medicine and biomedical device engineering, offering unprecedented opportunities to design materials that are both bioresponsive and transient. These materials, often engineered to respond to specific physiological stimuli such as temperature, pH, or electric fields, provide dynamic platforms for tissue regeneration, drug delivery, and the fabrication of implantable devices with controlled degradation profiles [5,6]. Unlike conventional permanent implants, biodegradable polymers naturally degrade into non-toxic byproducts, thereby reducing the need for surgical retrieval and minimizing long-term immune responses [7].

Smart polymers, particularly shape memory polymers (SMPs), have emerged as a promising class capable of undergoing reversible shape transformations under external stimuli, making them ideal for minimally invasive surgical procedures, vascular occlusion, and self-tightening sutures [8]. Their adaptability is further enhanced by functional integration with conductive or antimicrobial agents, enabling multifunctional performance such as localized drug release or bacterial resistance [9,10].

In tissue engineering, these materials enable the fabrication of scaffolds that not only mimic the mechanical and architectural features of native extracellular matrices but also actively guide cellular behavior. Techniques such as electrospinning, 3D printing, and emulsion-freezing allow precise control over pore morphology, mechanical integrity, and bioactive loading—factors essential for successful tissue integration and regeneration [11,12]. The application of additive manufacturing has further accelerated the customization of patient-specific implants and scaffolds, aligning with the broader goals of precision medicine [13].

Furthermore, hybrid materials that combine natural and synthetic polymers leverage the biological compatibility of biopolymers with the tunable mechanics of synthetic backbones. Such composites show enhanced hydrophilicity, degradation control, and cellular affinity, which are critical in cardiovascular stents, bone repair scaffolds, and septal occluders [14,15]. The incorporation of nanotechnology and bioactive ceramics, such as hydroxyapatite or bioactive glass, further promotes osteoconductivity and tissue integration, making these materials indispensable in orthopedic and dental applications [16,17].

Biodegradable polymers also hold relevance beyond traditional environmental and biomedical contexts, influencing a range of industrial sectors. For example, the study in [18] investigates the use of cross-linked polymeric compositions as temporary blocking agents in high-pressure well-killing operations. Although these materials are not biodegradable in the strict environmental sense, they exemplify how polymer degradation and controlled disintegration can be tailored for subsurface engineering applications—an approach conceptually aligned with the broader objectives of environmentally responsive and transient materials.

Smart (stimuli-responsive) materials are revolutionizing biomedical devices and therapies. By definition, smart polymers respond to external triggers (temperature, pH, light, etc.) with changes in shape, stiffness or permeability. In medicine, this enables dynamic devices: for example, a shape-memory stent that self-expands at body temperature, or a hydrogel that releases a drug in response to inflammation. Smart biomaterials “can alter their properties in response to external stimuli,” which is highly attractive for tailored biomedical functions [19]. For instance, SMPs have been engineered to meet specific surgical device requirements by modifying chemistry for biocompatibility and actuation profiles. As noted in recent reviews, such materials find applications across tissue engineering (e.g., scaffolds that adapt to tissue growth), drug delivery (on-demand release), and implantable devices (e.g., self-folding structures) [20]. In short, smart polymers add “on-the-fly” adaptability to biomedical designs—they support personalized dosing, reduce the need for invasive procedures, and can improve integration with dynamic biological environments [21].

This review highlights how the integration of smart material design with cutting-edge fabrication techniques is driving the advancement of next-generation biomedical devices and tissue-engineered systems. These emerging technologies are expanding the functional potential of implants and scaffolds while enabling minimally invasive, personalized, and biologically compatible solutions.

## 2. Relevance and Methodology

The convergence of smart and biodegradable polymer technologies has catalyzed significant progress in tissue engineering and interventional medicine. These advanced biomaterials offer unprecedented control over mechanical, chemical, and biological properties, making them ideal for scaffolds, drug delivery platforms, and implantable devices. By mimicking the extracellular matrix and responding to physiological stimuli, smart polymers facilitate targeted therapy, tissue regeneration, and minimally invasive interventions. This review synthesizes recent advancements in the design, fabrication, and clinical translation of polymeric systems, emphasizing their critical role in personalized medicine, regenerative strategies, and next-generation medical devices.

This review adopts a thematic approach, systematically analyzing recent literature on smart and biodegradable polymers with applications in tissue engineering and interventional devices. Sources were selected from peer-reviewed journals and indexed databases to ensure scientific rigor. The paper categorizes materials based on function—such as shape-memory behavior, conductivity, or biodegradability—and discusses fabrication techniques including electrospinning, freeze-drying, and emulsion-based processing. Emphasis is placed on the synergy between natural and synthetic polymers, nanocomposites, and manufacturing innovations like 3D printing. The methodology ensures a comprehensive overview of material performance, clinical relevance, and future research directions.

Table 1 contains a comparison of the current review to other recent reviews on this topic.

## 3. Trends and Advances in Smart and Biodegradable Materials

### 3.1. Shape Memory Polymers

**Figure 1:**
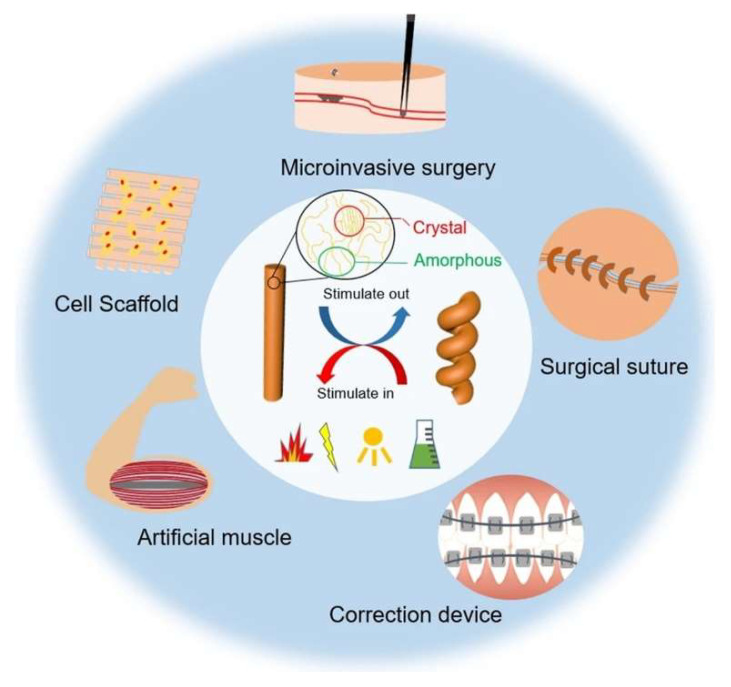
Illustrative summary of shape memory materials highlighting their activation mechanisms, types of external stimuli, and a range of current or prospective applications [30]. (The figure is available under Open Access).

Shape memory polymers (SMPs) are a class of polymers capable of holding temporary shapes and returning to their original form when exposed to specific external triggers such as temperature, chemical agents, pH changes, or light. This reversible transformation is typically governed by dynamic covalent bonding or supramolecular interactions. Various potential applications of SMPs are illustrated in Figure 1 [30].

Currently, adaptive medical devices such as clamps, staples, and clips—widely used in surgical procedures—are often fabricated from shape memory alloys (SMAs) like Nitinol due to their pseudoelastic and shape-memory effects [31]. However, metallic materials possess several well-documented drawbacks, including limited recoverable strain (typically < 8%) [32], risk of overstressing under heavy loads, and lack of bioresorbability. These limitations may be effectively addressed through the use of shape memory polymers (SMPs) and related composites. Compared to metals, SMPs offer several advantages: higher recoverable deformation (up to 400–800%), tunable shape-recovery temperatures, and customizable mechanical and physicochemical properties. A further notable benefit of SMPs is their potential for remote activation—without direct thermal stimulation—via external triggers such as laser irradiation, electrical currents, or magnetic fields [1].

The key advantage of sutures made from SMPs lies in their self-tightening capability. They can be loosely threaded and later tightened through activation of the shape memory effect, which is particularly beneficial in minimally invasive surgery where knot-tying is challenging [33].

Another advantageous application of SMPs is in drug delivery via cardiovascular stents. Conventional metal-based stents require additional fabrication steps to coat drugs onto polymer layers, increasing production costs. In contrast, drugs can be directly embedded into the SMP matrix, reducing complexity and cost [34,35,36].

Buffington and colleagues developed a shape memory polymer composed of poly(ε-caprolactone) (PCL) and pellethane. The shape memory effect was programmed using a series of thermomechanical cycles in which the polymer was heated above the PCL melting point and mechanically deformed [37].

Lee and co-workers developed electroactive, shape-memory polyurethane dressings capable of closing cracked or gaping wounds without sutures, highlighting their utility in wound care [38]. An excellent overview of the application of shape memory polymers (SMPs) for studying mechanobiology is provided in the review by Ebara [39].

SMPs are also being developed for treating endovascular thrombosis. Small and colleagues created a hybrid device combining nitinol and SMPs to remove blood clots in vitro [40].

Zhao et al. incorporated carbon nanotubes into SMPs to create an injectable, conductive, antibacterial shape memory material suitable for hemostasis. This injectable polymer adapts to irregular wounds and exerts pressure upon expansion, aiding in the treatment of blast-induced or non-compressible injuries [41].

Wan and co-authors developed SMP-based inks suitable for 3D printing, with demonstrated applicability for suture and stent fabrication [42].

Neffe et al. designed a biodegradable SMP-based ureteral stent that can be temporarily deformed and then fixed in vivo upon recovery to its permanent shape. By loading the polymer with drugs, such stents may treat various conditions, including cancer, kidney stones, or complications during pregnancy [43].

Finally, Song et al. demonstrated that SMP copolymers based on poly(dimethylsiloxane-co-ε-caprolactone) could trigger differentiation of embryonic stem cells by modulating substrate stiffness. They also observed that incorporating microspheres of their material into embryoid bodies led to size-dependent protein expression patterns [44].

**Figure 2:**
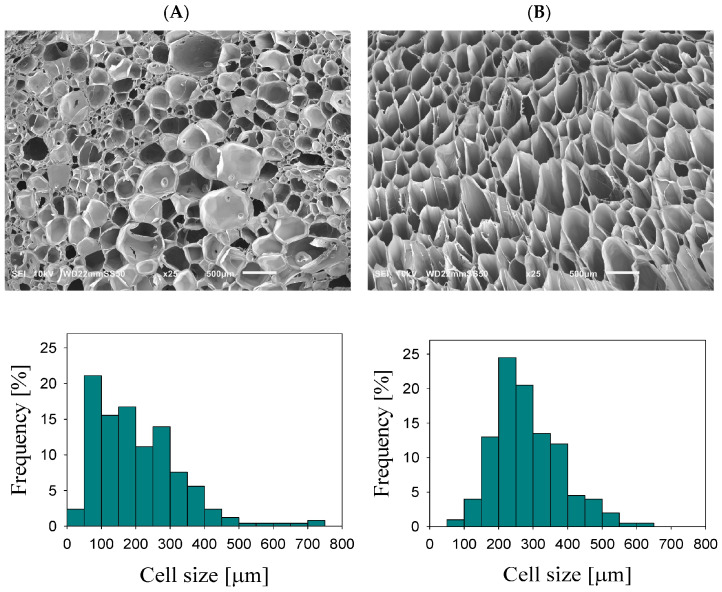
Scanning electron microscopy (SEM) images of foamed PLA/PA nanoblends (**A**) and in situ formed nanocomposites (**B**), accompanied by their respective cell size distribution graphs [45]. (The figure is available under Open Access).

Figure 2 presents SEM micrographs of the foamed PLA/PA nanoblends and the in situ generated nanocomposites, alongside their respective cell size distribution profiles. The foamed PLA/PA nanocomposite exhibits a uniform cellular morphology, characterized by a unimodal distribution and an average cell diameter of 280 μm. In contrast, the foamed PLA/PA nanoblend displays a more intricate structure, featuring a bimodal distribution comprising larger cells averaging 316 μm and smaller ones around 70 μm. The total cell density is marginally greater in the nanoblend foam (1.78 × 10^6^ cells/cm^3^) compared to the nanocomposite foam (0.88 × 10^6^ cells/cm^3^). It is important to consider, however, that this increased cell concentration in the nanoblend is influenced by the substantial number of small cells typically situated at the junctions of larger cells [45].

### 3.2. Tissue Engineering

Tissue engineering stands out as one of the most interdisciplinary and rapidly evolving fields. Scaffold materials and fabrication technologies are crucial in this area. A wide variety of polymer-based scaffolds, both natural and synthetic, have been employed in tissue engineering to date. Regardless of origin, scaffolds must meet specific design criteria to be functional and clinically valuable [46]. Each fabrication method offers unique benefits under different processing parameters, and recent innovations have improved scaffold properties, leading to the development of more effective implants. For example, electrospun nanofibers provide ECM-mimicking topography that enhances cell orientation and differentiation [47]. Ideally, scaffolds should be both surface-compatible and architecturally congruent with host tissues. Future development should aim at novel scaffold designs and a deeper understanding of their interactions in the biomedical context, especially when processed from smart polymers. Nanotechnological approaches can be particularly beneficial in engineering scaffold features at dimensions that are favorable for cellular and biomolecular interactions [48].

Common issues related to insufficient surface compatibility of biomaterials fall into four main categories.

- First, mechanical or topographical mismatch—if the material’s surface is too rigid or rough, it can damage surrounding tissue upon contact [49]. In the case of composite scaffolds, it is essential to ensure good phase compatibility while maintaining porosity and mechanical strength [50,51].
- Second, all artificial materials introduced into the body are at risk of biofouling, which frequently results in infection and eventual device failure [52]. Biodegradable polymers, especially when combined with bioceramics, generally provoke a milder inflammatory response [53].
- Third, direct immune system responses to implant materials can cause complications. These may arise from active immune attacks or from fibrous encapsulation that chronically separates the implant from tissue [54,55].

Hybrid biomaterials, especially those combining synthetic and natural polymers, offer improved hydrophilicity, cellular adhesion, and biodegradability. Common synthetic polymers in tissue engineering include polyglycolide (PGA), polylactide (PLA), poly(lactide-co-glycolide) (PLGA), poly(D,L-lactic acid) (PDLLA), polyethylene glycol (PEG), and PCL. These can be modified or self-reinforced to improve mechanical strength [56]. Hybrid biomaterials—created by combining organic and inorganic components—offer multifunctionality and tailored thermal, structural, and mechanical stability [57].

Polypyrrole (PPy), another intrinsically conductive polymer, has also been extensively explored in biomedical and tissue engineering contexts [58,59,60,61]. PPy can be chemically modified and combined with other polymers to create biocompatible electroactive environments that support cellular growth and communication. Such scaffolds can enhance cell viability, proliferation, and intercellular interaction, increasing the likelihood of directed differentiation toward a cardiomyocyte-like phenotype. For example, electrospun 3D scaffolds based on poly(lactic-co-glycolic acid) (PLGA)/PPy have demonstrated favorable biocompatibility with murine cardiac progenitor cells and human-induced pluripotent stem cells (hiPSCs). Notably, hiPSCs were observed to align and proliferate along conductive fibers for up to 10 days without significant apoptosis [62].

A broad spectrum of both natural and synthetic inorganic biomaterials—ranging from metals to ceramics—has been developed for the restoration or replacement of damaged musculoskeletal and periodontal tissues. These materials are employed in orthopedic implants, bone grafting, dental prosthetics, and cement applications [63].

SMPs typically lack intrinsic antibacterial activity, which can be conferred by incorporating antimicrobial agents. Toncheva et al. embedded silver nanoparticles grafted onto cellulose nanocrystals into a PCL-based SMP network, producing an IR-responsive SMP (780–1400 nm, 150 W) with antimicrobial properties. The composite exhibited a minimum inhibitory concentration (MIC) of 16 μg/mL against Gram-positive bacteria and achieved shape recovery of ~90%, making it suitable for biomedical applications like self-tightening sutures [64].

### 3.3. Degradable Synthetic Polymers Drug Delivery and Bone Repair

The main incentive to use biodegradable synthetic polymers lies in their strength and rigidity during bone regeneration. However, erosion and acidic by-products can pose risks of premature scaffold collapse and inflammation. These issues can be mitigated by tuning molecular weight, chemistry, and crystallinity [65,66].

Electrospinning is a commonly used method for fabricating polymer-based nanofibrous scaffolds. This technique can produce fibers ranging from nanometers to micrometers in diameter, depending on the characteristics of the polymer solution [67,68].

Recently, biodegradable polymers such as polylactic acid (PLA), polydioxanone (PDO), polycaprolactone (PCL), polyglycolic acid (PGA), and poly(lactic-co-glycolic acid) (PLGA) have gained significant attention. These materials are known for their excellent biocompatibility and bioresorbability, making them highly suitable for medical applications such as implants, coronary stents, drug delivery, tissue engineering, and heart valves [69,70,71].

Biodegradable polymers such as PLA, PGA, and PCL are widely used in bone tissue engineering, often in combination with bioactive materials like hydroxyapatite (HA), β-tricalcium phosphate (β-TCP), and bioactive glass (BG). These scaffolds are also employed in clinical settings when loaded with growth factors due to their strong bone regenerative capacity. However, their broader application is often limited by high manufacturing costs. A notable example of a metallic matrix composite is Ti6Al4V [72,73].

PLA is thermally stable, cytocompatible, and decomposes into nontoxic byproducts. Its forms—PLLA and PDLA—can be blended to optimize degradation rates [74]. PLGA, a copolymer of PGA and PLA, is FDA-approved for its controlled degradability and biocompatibility [75]. PCL, another FDA-approved polymer used in bone repair, features a low melting point, good miscibility, and excellent blend compatibility [76,77,78].

Poly(acrylic acid) (PAAc) has been widely employed in bioadhesive drug delivery systems. Polymers are essential components in achieving controlled drug release, which is critical in applications such as transdermal patches, microspheres, pumps, aerosols, ocular implants, and contraceptive devices. The mechanisms of drug release from these systems generally follow three primary pathways [79,80].

Polymeric micelles (PMs) have emerged as multifunctional nanocarriers with significant promise in drug delivery systems. They can enhance drug solubility, control release profiles, and improve drug accumulation at target sites through the enhanced permeability and retention (EPR) effect. PMs are especially suitable for poorly water-soluble chemotherapeutics due to their excellent biocompatibility, minimal toxicity to healthy cells, and capability to solubilize a wide range of drugs in their hydrophobic core. These characteristics make PMs a compelling option in future cancer therapies [81].

Poly(diol citrate) elastomers (often known as citrate-based polymers) constitute a versatile and important class of biodegradable materials synthesized via polycondensation of citric acid with aliphatic diols—typically 1,6 hexanediol, 1,8 octanediol, 1,10 decanediol, or longer. Unlike many other systems, they form cross-linked networks without external catalysts and at relatively low temperatures, sometimes as mild as 37 °C [82].

These materials display tunable mechanical properties akin to native soft and hard tissues, with reported tensile strengths up to ~11 MPa, Young’s modulus spanning 1.6–14 MPa, and elongations exceeding 500%, depending on diol choice and curing conditions [83]. Their mechanical adaptability and resilience render them particularly relevant for orthopedic and tissue engineering scaffold applications.

A critical innovation has been their integration with hydroxyapatite (HA) to create composites better mimicking bone composition. Poly(diol citrate)/HA composites can incorporate up to ~60–65 wt% HA—substantially more than PLA-based systems—thanks to the calcium chelating carboxyl groups inherent in the citrate moiety [84]. These composites exhibit excellent osteoconductivity, accelerated mineral deposition, and minimal chronic inflammation when implanted in vivo.

Mechanical testing of POC-HA composites (65 wt% HA) shows compressive strength ≈ 41 MPa and flexural strength ≈ 9.7 MPa, comparable to commercial fixation devices, while maintaining significant elasticity. Simulated body fluid immersion induces abundant mineralization (Ca/P ratio = 1.5–1.7), and in vivo rabbit knee implants demonstrate osteoconductivity and biocompatibility [85].

Synthetic polymers such as poly(L-lactic acid) (PLLA), poly(D,L-lactic-co-glycolic acid) (PLGA), polystyrene, and polyglycolic acid (PGA) are widely used in scaffold production. Their degradation rates can be fine-tuned by altering the polymer composition. However, their relatively low bioactivity poses a risk of tissue rejection [86,87].

**Figure 3:**
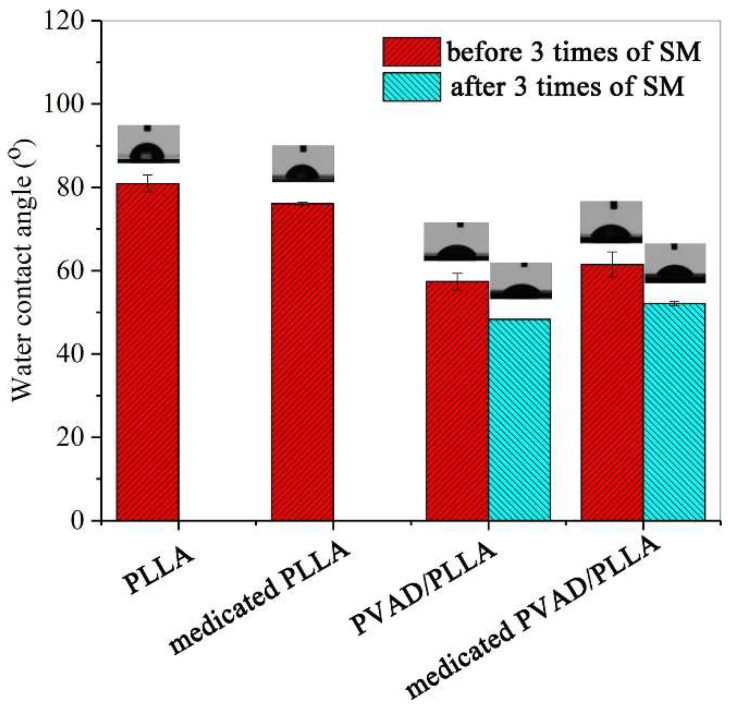
Water contact angle measurements of PLLA, drug-loaded PLLA, PVAD/PLLA, and drug-loaded PVAD/PLLA samples, both prior to and following three SM cycles [88]. (Permission for use was granted by Elsevier).

Wettability significantly influences both moisture interaction and drug release behavior. As shown in Figure 3, incorporating salicylic acid into PLLA reduces its water contact angle from 80.9° to 76.1° due to the drug’s hydrophilic nature. Blending PVAD (polyvinyl alcohol derivative) with PLLA further lowers the angle to 57.4°, indicating increased surface hydrophilicity. However, when salicylic acid is added to PVAD/PLLA, the contact angle slightly increases to 61.5°, likely due to hydrogen bonding between PVAD and the drug, which limits the availability of hydrophilic groups—suggesting drug encapsulation by PVAD. After three SM (shape memory) cycles, the rougher, more porous PLLA structure exposes additional carboxyl groups, further reducing the contact angle to 52.1° in medicated PVAD/PLLA samples [88].

To replicate the description of bone—which is itself a natural composite—inorganic–organic materials present improved mechanical and biological properties compared to their single components. Optimal proportions are essential to promote bone formation while preserving porosity and scaffold integrity. A vast range of combinations has yielded composites suitable for bone tissue engineering [89,90,91,92,93].

Artificial tendons and ligaments are commonly fabricated from polyethylene terephthalate (PET) [94] and polyurethane (PU). Polyvinyl chloride (PVC) is perhaps the most prevalent material in medical tubing, including endotracheal tubes, catheters, and blood lines [95].

High-performance thermoplastics such as polyether ether ketone (PEEK) are also employed in load-bearing implants, such as intervertebral disc replacements [96] and even in dental implants [97].

Silicone rubbers have gained popularity in biomedical applications primarily due to their bioinert properties. Examples include small joint prostheses, breast implants, and medical tubing. Some silicones, such as polydimethylsiloxane (PDMS), are highly transparent and are used in contact lenses or endoscopic windows. Biodegradable polymers are also commonly used as coating materials. For example, polyvinyl alcohol (PVA) hydrogels and polycaprolactone (PCL) scaffolds are often coated with biopolymers to enhance their lubricity, adhesion, and hemostatic properties [98].

**Figure 4:**
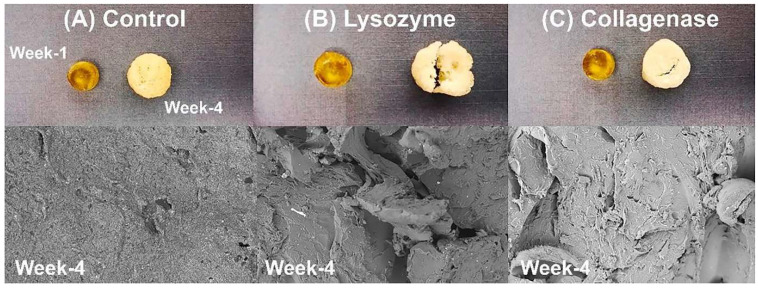
Digital and scanning electron microscopy images of hydrogels depicting structural alterations after four weeks [99] (permission for use was granted by Elsevier).

Hydrogels can retain significant amounts of water and biological fluids due to their intricate, hydrophilic 3D network. They degrade through various mechanisms such as hydrolysis, erosion, solubilization, and enzymatic breakdown, all of which are influenced by environmental conditions and the polymer’s properties, including its origin and ionic interactions [99]. For instance, gelatin is particularly susceptible to enzymatic degradation by collagenase and lysozyme. Figure 4 illustrates the resulting weight loss and structural changes.

In bone defect repair, Zhai et al. fabricated composite scaffolds of poly(N-acryloylglycinamide) and nanoclay that stimulated osteogenic differentiation and enhanced bone regeneration [100]. Wang et al. printed SMP-based scaffolds incorporating superparamagnetic iron oxide nanoparticles to induce osteogenesis. Their ink formulation also contained gelatin and PEG for enhanced printability, yielding constructs with superior structural recovery and fixation. PEG presence further enhanced osteogenic outcomes [101].

High-performance polymers are a class of materials distinguished by outstanding mechanical properties—such as polyetheretherketone (PEEK) and polyetherketoneketone (PEKK) with a Young’s modulus of 3–4 GPa and tensile strength of approximately 100–120 MPa, and polyetherimide (PEI) with a modulus around 3 GPa and tensile strength of 150–160 MPa. In addition to their excellent chemical resistance and high continuous service temperatures, these polymers are significantly more expensive than engineering-grade alternatives [102].

Over recent decades, biocompatible polyurethanes (PUs) have gained considerable attention in the biomedical field due to their tunable properties and adaptive performance. Medical-grade PU formulations are available under various trade names, including Carbothane, Pellethane, and Tecoflex from Lubrizol (USA), and Carbosil and Bionate from DSM (Netherlands). Derived primarily from fossil-based sources, these PUs have established a strong presence in the medical community, earning the trust of surgeons and clinicians. Typically thermoplastic and occasionally blended with other polymers, these materials can be tailored for specific biomedical applications [103,104,105].

Polyurethanes are currently among the most commonly used polymers in biomedical devices due to their high biocompatibility and hemocompatibility. These properties make them ideal candidates for use in blood-contacting medical products such as vascular catheters, breast implants, blood bags, heart valves, and vascular grafts [106,107,108]. Polyurethanes, as well as their blends and composites, are being widely investigated for use in shape-memory biomaterials [108,109,110,111] and as drug delivery matrices [112,113,114,115].

Polymeric materials also play a crucial role in skin tissue regeneration research. Among synthetic options, biocompatible polyurethane (PU) is frequently used [116,117,118], alongside polylactic acid (PLA) [119], polycaprolactone (PCL) [120,121], and polyglycolide (PGA) [122].

Phosphorylcholine (PC), a zwitterionic compound naturally present on the outer membrane of red blood cells, carries both positive and negative charges in balanced amounts within a single molecule. Due to this unique structure, SPUs incorporating PC are anticipated to possess excellent blood compatibility, making them promising materials for biomedical applications [123].

**Figure 5:**
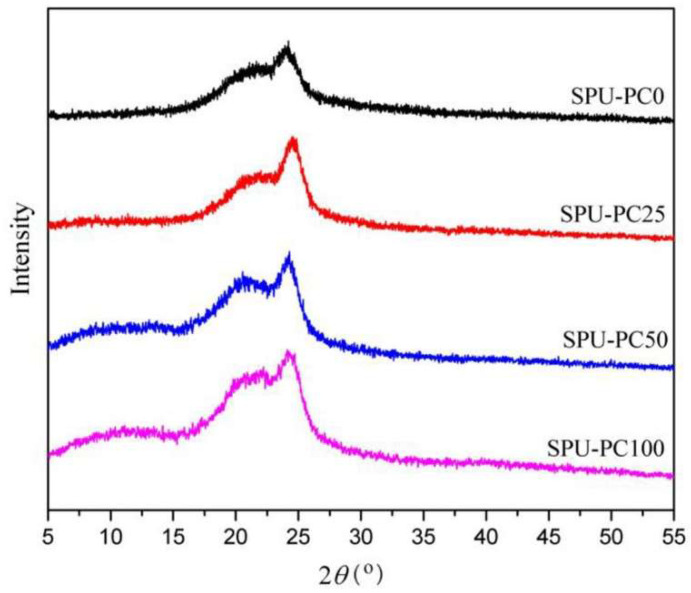
X-ray diffraction profiles of SPU-PC films containing different amounts of phosphorylcholine [124]. (Permission for use was granted by Elsevier).

In [124], the authors prepared and characterized segmented poly(ester-urethane) (SPU) with PC. XRD analysis was performed to study the crystallization behavior of SPU-PC films with varying PC content, as shown in Figure 5. All samples displayed two broad diffraction peaks at around 21.4° and 24.2°, corresponding to crystalline regions in the soft and hard domains, respectively—indicating a clear micro-phase separation. As the PC content increased from SPU-PC0 to SPU-PC100, the intensity of these peaks also rose, suggesting higher overall crystallinity. This was attributed to more organized hard domains due to uniform hard segment distribution and the formation of new hydrogen bonds between PC side chains and carbamate groups, enhancing both micro-phase separation and soft segment crystallization. Additionally, a weak broad diffraction feature between 7° and 13° appeared in SPU-PC50 and SPU-PC100, likely resulting from the slight crystallization of polar PC groups. These findings aligned with the DSC results.

Poly(L-lactic acid) (PLLA), an FDA-approved biodegradable polyester, is extensively used in biomedical applications such as drug delivery systems, tissue engineering, and medical devices, owing to its semicrystalline structure [125,126,127].

The first is the diffusion-controlled mechanism, where the drug is released through dissolution and subsequent diffusion across the polymer matrix. The second is the erosion-controlled mechanism, which involves drug release triggered by polymer degradation, dissolution, or disintegration. The third mechanism is based on osmotic control, where water uptake from the surrounding environment governs the drug release rate [128].

Smart polymers have found extensive use in drug delivery and bioseparation applications. Mimicking biological systems, these polymers exhibit remarkable adaptability and performance in aqueous environments [129].

Most smart polymers are stimuli-responsive in aqueous media. A notable example is hydrogels, which, while structurally similar to linear polymers, form three-dimensional networks capable of swelling in water without dissolving. Their responsiveness to environmental stimuli makes them highly effective in bioseparation processes due to their capacity for volume change under specific conditions [130].

Changes in pH or temperature can induce swelling or contraction of hydrogel granules. These transitions are key to controlled drug release, as the diffusion of therapeutic agents is highly dependent on the hydrogel’s physical state [131].

Polymers are also essential in vascular prosthetics and systems requiring unobstructed blood flow. Materials used include polyethylene terephthalate fibers, expanded polytetrafluoroethylene foams, segmented porous polyurethanes, and microporous silicone rubber [132]. In addition, polymers are critical for blood oxygenation, necessitating hemocompatibility. Polypropylene, both in solid and microporous forms, has been utilized during cardiopulmonary surgeries.

### 3.4. On-Demand Degradable Polymer

On-demand degradable polymers are engineered to remain inert during use and then to break down rapidly into small fragments when a specific trigger is applied [133]. This controlled degradability is valuable both for sustainability (facilitating recycling and reducing persistent waste) and for advanced biomaterials—for example, enabling “self-erasing” implants or triggered drug-delivery systems [134]. Indeed, backbone-degradable polymer networks have attracted interest in drug delivery and tissue engineering contexts [135].

These polymers typically incorporate cleavable linkages that respond to defined stimuli. Common triggers include light (via photolabile bonds), extreme pH (acid- or base-sensitive bonds), redox agents (e.g., disulfide/diselenide linkages cleaved by oxidative/reductive conditions), specific enzymes, and in some cases, heat or mechanical force. For instance, a pH drop can hydrolyze acetal or ester crosslinks, certain enzymes can cut peptide or polysaccharide segments, and cellular reductants (like glutathione) can sever disulfide bridges—all yielding on-demand polymer degradation [136].

On-demand degradable polymers have found a range of biomedical uses. For example, calcium-alginate microspheres have been developed as imageable embolic agents: they can occlude a blood vessel during a procedure and then rapidly dissolve on command when an injected trigger (an ion chelator) is applied, restoring blood flow [137]. Likewise, “smart” hydrogels have been created for controlled drug release: one poly(ethylene glycol)–polydopamine hydrogel, crosslinked via diselenide bonds for diabetic wound therapy, was shown to remain stable until exposed to oxidative (H_2_O_2_) or reductive (thiol) stimuli, which cleaved the Se–Se crosslinks and caused the gel to disassemble [138]. This hydrogel exhibited precisely on-demand degradability and controlled release of its payload upon triggering. In these ways, on-demand polymers enable implant scaffolds, drug carriers, and coatings that vanish or de-bond only when and where needed.

A major challenge is that many commercial polymers have inert C–C backbones that resist cleavage, so novel chemistries are required to introduce triggerable bonds without compromising material performance [139]. Looking forward, researchers are expanding the “toolbox” of responsive linkages and exploring multi-stimuli designs. For example, polymers that respond to both pH and light or to biological and mechanical cues are under development. Such advances—along with a focus on biocompatible, bio-based materials—should broaden the utility of on-demand degradable polymers in medicine and beyond [140].

### 3.5. Aniline-Based Biomaterials

Oligoaniline-based conductive biomaterials have emerged as promising candidates for fabricating scaffolds tailored to support tissue regeneration by emulating key biological functions. Their conductivity can be fine-tuned to match physiological levels observed in tissues and cells (10^−8^–10^−3^ S/cm) [141,142]. These materials can be processed into various architectures—including nanofibers [143], hydrogels [144], and particles [145]—using diverse fabrication strategies. Oligoaniline-based materials have found applications in tissue scaffolding, drug delivery [146], neural probes [147], and biosensors [148].

Recent studies have demonstrated the benefits of integrating conductive polymers—either with or without electrical stimulation—into scaffolds to promote enhanced tissue regeneration in a range of applications, including neural [149], cardiac [150], bone [151], and liver tissue engineering [152,153,154]. Conductive polymers have shown promise in promoting chronic wound healing [155] and in enhancing endochondral ossification [156]. Certain conductive biomaterials, such as carbon nanotubes (CNTs) [157] and conductive polymers, are capable of modulating cellular morphology and growth [158].

While various conductive polymers have been utilized in tissue engineering, oligoaniline-based biomaterials have gained more attention due to their ease of synthesis and lower cost relative to pyrrole and thiophene oligomers. These low-molecular-weight oligomers can be incorporated into polymer structures either by grafting to the polymer backbone or acting as co-monomers. The incorporation strategies generally fall into two categories: (1) grafting oligoanilines onto biocompatible polymers, such as the aniline pentamer-chitosan (CS-AP) system [159], and (2) forming block copolymers by integrating oligoanilines into the polymer backbone, such as the PLA-aniline pentamer (PLA-AP) system [160].

Compared to other conductive polymers, aniline-based oligomers are biodegradable and are metabolized by macrophages or eliminated via renal clearance [161]. Kashi and colleagues developed thermosensitive oligo-pyrrole/chitosan (CS) hydrogels for cardiac tissue engineering that exhibited both biodegradability and biocompatibility [162]. Additionally, pyrrole oligomers have been explored for neural regeneration [163] and DNA targeting [164]. Spicer et al. recently introduced a thiophene oligomer-based platform as a scaffold candidate for tissue engineering [165].

Both natural and synthetic biomaterials—including cardiac patches [166], injectable hydrogels [167], electrospun nanofiber composites [168], nanoparticles [169], and 3D hydrogel constructs [144]—have been investigated to replicate the mechanical properties of the cardiac extracellular matrix and restore myocardial function [170,171].

Polyaniline (PANI) is among the most widely studied conductive polymers in tissue engineering due to its intrinsic conductivity and biocompatibility [172,173,174,175,176]. Its electrical properties can be adjusted through chemical or electrochemical doping, such as p-type (oxidation) or n-type (reduction) doping [177].

**Figure 6:**
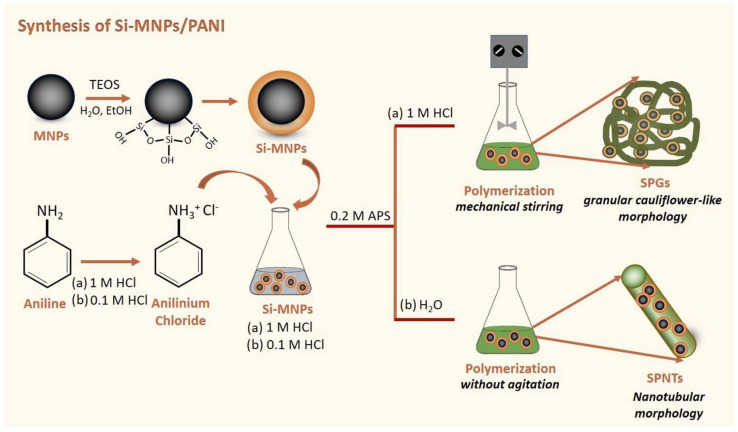
Diagrammatic overview illustrating the synthesis process of Si-MNPs/PANI nanocomposite materials [173]. (Permission for use was granted by Elsevier).

The authors of [173] show the creation of innovative nanocomposites that respond to dual external stimuli, designed for biomedical use. These composites, composed of silica-coated iron oxide and polyaniline (Si-MNPs/PANI), were synthesized through the oxidative polymerization of aniline in the presence of Si-MNPs at concentrations of 25% and 50% by weight. By adjusting factors like acid concentration and mixing technique during synthesis, the resulting Si-MNPs/PANI composites were produced in two distinct morphologies: nanotubes (SPNTs) and granules (SGTs). Si-MNP/PANI nanocomposites were synthesized through in situ polymerization of aniline in the presence of silica-coated magnetic nanoparticles (Si-MNPs), as illustrated in Figure 6. To produce nanotubular structures (SPNTs), Si-MNPs (25 or 50 wt%) were mixed with aniline in 0.1 M HCl, followed by the addition of ammonium persulfate (APS) and left undisturbed in an ice bath for 18 h. For granular structures (SPGs), the same components were combined in 1 M HCl and stirred in an ice bath for 6 h. The resulting materials were then thoroughly washed with deionized water and ethanol and dried under vacuum at 60 °C for 12 h.

Conductive scaffolds based on poly(3,4-ethylenedioxythiophene), also known as PEDOT, represent excellent electrical interfaces with biological cells and tissues due to their inherent electrical conductivity, mechanical robustness, biocompatibility, and stability. These properties make them highly promising for tissue engineering applications [178,179,180].

### 3.6. Inorganic Biomaterials

Metallic biomaterials, such as titanium and its alloys, offer high strength, low modulus of elasticity, and low density. Meanwhile, ceramic biomaterials—or bioceramics—including aluminum oxide, zirconia, calcium phosphates, calcium phosphate cements (CPCs), and silicates, are known for their biocompatibility, osteoconductivity, and osteogenic potential [181,182].

Inorganic biomaterials can be categorized as bioinert, bioactive, or bioresorbable, based on their ability to form direct bonds with native tissues following implantation [183].

- Bioinert materials—such as aluminium oxide, zirconia, titanium, and its alloys—do not chemically interact with surrounding tissue. They are typically employed in load-bearing implants, for instance, bone-support devices and hip prosthesis femoral heads.
- Bioactive materials—like bioglasses and glass-ceramics—form direct bonds with living tissue and have been used to fill minor bone defects and periodontal irregularities.
- Bioresorbable materials—including calcium phosphates (CaPs), calcium phosphate cements (CPCs), calcium carbonates, and calcium silicates—undergo gradual resorption in vivo, eventually being replaced by natural bone.

Naturally derived inorganic biomaterials sourced from marine shells, coral, sponges, nacre, or animal bone (e.g., fish, poultry) provide rich calcium-based compounds (e.g., calcium carbonate, calcium phosphate) for biomedical use.

Synthetic inorganic biomaterials—encompassing alumina, zirconia, bioactive glasses and glass-ceramics, and CaP-based ceramics, coatings, and cements—are widely used in tissue engineering and regenerative medicine (TERM) [184,185].

Functionalization via Ionic Doping

Numerous studies have explored doping bioactive inorganic materials with therapeutic ions (Sr, Zn, Mg, Mn, Si). These ions are released gradually during bone resorption and can enhance implant biocompatibility and mechanical integrity:

- Sr/Mg/Zn-doped brushite cements [186].
- Si-substituted nanocalcium phosphates with improved osteoblast response [187,188].
- Ag-doped CaP ceramics for antimicrobial coatings [189].
- Mg-substituted apatites [190].
- Ionic-doped CaP/silk fibroin composites for bone scaffolds [191].

For example, in the study [192], poly(L-lactide) (PLLA) was combined with newly developed Ca/Mn co-doped BaTiO_3_ (CMBT) nanofibers to create substrates with enhanced piezoelectric performance following polarization. Unlike undoped BaTiO_3_ nanofibers, the CMBT variant not only retained piezoelectric functionality but also demonstrated improved bone-regenerative potential due to the bioactivity of the incorporated calcium and manganese ions.

**Figure 7:**
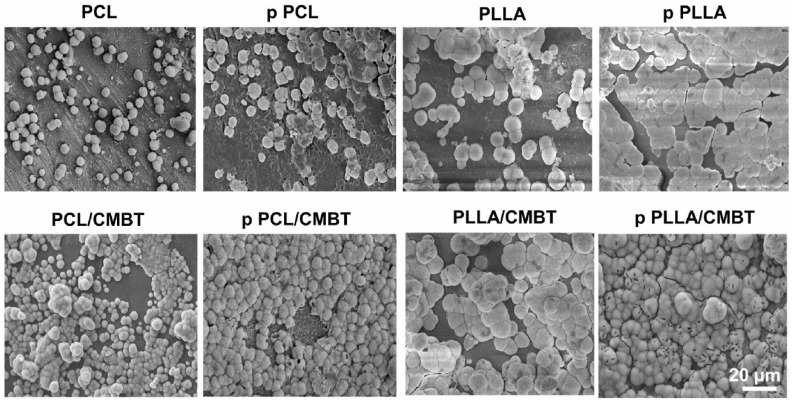
SEM micrographs illustrating the surface features of polarized and non-polarized membranes following a 7-day immersion in 1.5× simulated body fluid (SBF) [192]. (Permission for use was granted by Elsevier).

To assess this, membranes with and without polarization were immersed in 1.5SBF for seven days, and SEM analysis was conducted. As shown in Figure 7, non-poled PLLA and PCL, as well as polarized PCL membranes, exhibited minimal mineral formation. In contrast, polarized PLLA showed notably higher mineral deposition. Incorporating CMBT nanofibers into the PCL matrix further improved its mineralization ability, especially after polarization. The polarized PLLA/CMBT membrane demonstrated the highest mineral accumulation, aligning with its elevated d_33_ values and Zeta potential.

The 3D Scaffolds in TERM

Responding to the need for advanced TERM platforms, 3D scaffolds are designed to provide [193]:

1. Nutrient transport to support cell adhesion, proliferation, and differentiation,
2. Structural cues for cell attachment, growth, and migration,
3. Mechanical stability,
4. Controlled degradation without toxicity or inflammation.

Applications of Inorganic Biomaterials

Inorganic materials—particularly metals and bioceramics—are major players in bone repair and regeneration fields such as bone grafts, cementing agents, load-bearing prostheses (e.g., acetabular cups), and periodontal treatments [194].

Metallic biomaterials (e.g., titanium alloys) are valued for high strength, low elasticity modulus, and low density,

Bioceramics offer superior biocompatibility, osteoconductivity, and corrosion resistance [195,196].

Calcium phosphates (hydroxyapatite and tricalcium phosphate) mimic native bone mineral composition and have been widely used in bone repair [17].

Alumina and zirconia ceramics are also commonly used in orthopedic implants (hip and knee replacements) due to their chemical inertness, high strength, hardness, fracture resistance, and corrosion resistance [197,198].

Combining zirconia and alumina creates ZTA composites, improving fracture toughness, wear resistance, and chemical resilience—thereby reducing impact and dislocation risks while enhancing implant stability [199].

Bioactive glasses exhibit faster bonding to connective tissue versus typical bioceramics, forming an amorphous calcium-phosphate or hydroxyapatite layer upon implantation [200,201].

Gold is used in both pure and alloyed forms for medical applications such as dental fillings, middle ear reconstruction, pacemakers, and in vivo microchips [202]. Platinum, due to its excellent biocompatibility, corrosion resistance, and electrical conductivity, is ideal for electrodes in pacemakers, defibrillators, and neural devices like brain stimulators and cochlear implants [203].

Silver, known for its strong antimicrobial properties, is commonly used in nanoparticle form in medical applications. It can also be found as coatings or structural components in surgical instruments and bone replacements [204].

Ceramics, recognized for their superior mechanical strength and corrosion resistance, have been widely adopted as implant materials [205].

### 3.7. Natural Polymers

Natural polymers derived from renewable resources such as algae, plants, animals, and microorganisms closely resemble biological macromolecules and are readily recognized by the body’s environment [206]. Due to their structural similarity to the extracellular matrix (ECM), these natural polymers—commonly referred to as biopolymers—are less likely to trigger chronic inflammatory toxicity or immunological reactions compared to synthetic alternatives. As a result, they are particularly promising for the development of therapeutic systems, serving as bioactive agents and drug delivery platforms, as well as in the engineering of functional tissues.

Clinically utilized biopolymers for implant fabrication include proteins such as silk fibroin, collagen, gelatin, keratin, fibrinogen, elastin, and actin; polysaccharides such as chitosan, chitin, alginate, gellan gum and their derivatives; and glycosaminoglycans like hyaluronic acid [207]. Structural proteins such as elastin, fibrin, silk, and albumin have been applied in sutures, scar formation, and drug delivery systems [208,209].

Marine-derived collagen has recently gained interest as an alternative to mammalian collagen due to its cost-effectiveness and the abundance of marine waste material from which it can be extracted [210]. Although it can be used to fabricate polymer-based scaffolds without porogens, its inherent small and irregular pore structure limits broader applications [211].

Natural polymers such as collagen, gelatin, chitosan, agarose, alginate, and hyaluronate are commonly used in hydrogel synthesis to provide an ECM-like environment. However, their mechanical weakness, uncontrolled degradation, and immunogenicity remain major limitations [212,213,214]. These challenges are often addressed by combining natural polymers with synthetic ones such as poly(vinyl alcohol) (PVA), poly(2-hydroxyethyl methacrylate) (PHEMA), or poly(ethylene oxide) (PEO) [215]. The most frequently used natural materials in scaffold fabrication include collagen, alginate-based substrates, chitosan, and proteoglycans. These materials are biodegradable and assist host cells in producing ECM [216,217].

Cryogels—macroporous networks formed by thawing frozen polymeric materials—offer tunable porosity and mechanical characteristics. Their properties can be further enhanced by incorporating composite fillers and fibers [218,219].

Hydrogels, composed of highly hydrophilic three-dimensional crosslinked networks, are widely used to mimic ECM-based scaffolds. These materials can absorb up to 1000 times their dry weight in water without dissolving [220]. Their biocompatibility and structural tunability make them ideal for tissue engineering [221,222].

Natural and synthetic polymers offer complementary advantages, and their combination can yield materials superior to each class alone. Natural biomolecules (e.g., collagen, gelatin, chitosan) inherently provide bioactivity and cell-adhesion sites, while synthetic polymers (e.g., PLGA, PCL) supply mechanical strength and controlled degradation. By blending or co-fabricating them, scaffolds can achieve bone-like mechanics and biofunctionality simultaneously. For example, composite polymers have been engineered to mimic bone extracellular matrix: a natural–synthetic hybrid can yield strength, stiffness and fracture resistance close to natural bone, while remaining biodegradable [223]. Such composites also exhibit improved cell compatibility. In practice, combining collagen with PLLA or hydroxyapatite produces scaffolds whose strength and biocompatibility are optimized for bone repair. Thus, the synergy lies in leveraging natural polymers’ bioactivity with synthetic polymers’ tunable mechanics—a strategy explicitly reported to enhance scaffold properties and tissue regeneration [47].

Traditional polymerization approaches include step-growth and free-radical (chain-growth) methods. For example, biodegradable polyesters like PLA or PCL have long been produced by melt polycondensation or ring-opening polymerization (ROP) of cyclic monomers [224]. These methods are robust but yield relatively broad molecular-weight distributions and less control over architecture. In contrast, modern controlled/“living” polymerizations (e.g., ATRP—Atom Transfer Radical Polymerization, RAFT—Reversible Addition–Fragmentation Chain Transfer) and ROP with precise catalysts allow tailored architectures. For instance, combining ROP and ATRP (a reversible-deactivation radical polymerization, RDRP) has enabled well-defined biodegradable polymers with narrow dispersity [225]. Likewise, “click” chemistry and enzyme-catalysis are emerging as advanced routes for smart polymer synthesis. Overall, traditional methods (polycondensation, dispersion polymerization, ROP under simple catalysts) are still used for bulk polymer production, whereas advanced techniques (ATRP/RAFT, “green” ROP, photopolymerization, etc.) allow precise control of molecular weight, branching, and functional end-groups, yielding polymers optimized for smart behavior [226]. In summary, modern polymerization enables the design of stimuli-responsive biopolymers with predictable properties, while classical methods remain workhorses for bulk biodegradable polymer production [227].

## 4. Mechanisms and Design Principles of SMPs

Shape-memory polymers (SMPs) are “smart” polymer networks that can be deformed into a temporary shape and later recover a predetermined permanent shape in response to an external stimulus. In SMPs, the polymer matrix is engineered with two distinct phases: a permanent (hard or crosslinked) phase that defines the original shape and a reversible (soft or switchable) phase that fixes a deformed shape at lower temperatures. Upon heating above a transition temperature (typically the glass transition, Tg, or melting temperature, Tm of the soft phase), the network softens and can be deformed; cooling then “locks in” the temporary shape, and reheating causes recovery to the permanent shape [228]. Because SMPs respond to a variety of stimuli (heat, light, pH, moisture, etc.), designs can be tuned for specific biomedical environments. For example, incorporating chemical crosslinks or crystalline domains creates the permanent network, while an amorphous polymer segment serves as the switching phase [229]. Typical SMP matrices include biocompatible polymers such as polyurethanes, poly(ε-caprolactone), or polylactide, often blended or copolymerized to achieve the desired switching transition near physiological conditions.

SMPs can be triggered by many stimuli. In biomedical contexts, the most common trigger is heat, but others include light and local chemical changes. For example, water- or pH-triggered hydrogels can change shape when the local pH shifts. In general, SMPs can respond to thermal, photonic, electrical, mechanical, magnetic, or chemical (pH) triggers [230]. This stimulus flexibility is a key design principle: one can embed photo- or thermo-sensitive moieties (e.g., metal nanoparticles, carbon nanotubes, or azobenzene groups) to tailor which stimulus is effective. Because biomedical use demands biocompatibility, SMP designs often leverage inherently biocompatible or biodegradable polymers and fillers. In practice, polymer blends or copolymers are chosen so that the actuation threshold (for example, Tg lies just above body temperature. In one example, adding a low-weight plasticizer (oligomeric lactic acid) to PLA/TPS blends lowered the polymer’s Tg to about 45 °C, enabling full shape recovery at near-physiological temperatures [231,232]. Importantly, the design must maintain two well-separated phases—one phase must remain fixed (permanent) while the other undergoes transition—since loss of this two-phase morphology abolishes the shape-memory effect [233]. (Rigid crosslinks or crystals provide the “permanent” network, while amorphous segments act as the “switchable” domain [234]. In summary, key SMP design principles for biomedical use include (1) a dual-phase polymer network (permanent vs. reversible domains) [235], (2) tuning the thermal transition to a safe range (near 37 °C) by copolymer or plasticizer (as shown with PLA blends [236], and (3) embedding functional groups or additives (e.g., nanoparticles, ionic groups) to enable remote or specific stimulus response.

### 4.1. Thermally Activated SMPs

Thermally-induced SMPs are the most established class. In these systems, the polymer’s switching phase has a melting or glass transition that serves as a “switch”. In practice, one heats the SMP above its Ttrans (usually the Tg of the soft segment), deforms it to a temporary shape, and cools it to fix that shape; reheating above Ttrans then allows chain mobility and spontaneous recovery of the permanent shape [237]. Because many biomedical implants or scaffolds must actuate around body temperature, SMPs for medicine are often designed with Tg near 40 °C. For example, Sessini et al. showed that blending PLA with an oligomeric lactic acid plasticizer lowered Tg to ~45 °C, enabling shape recovery just above physiological temperature [238]. Without such tuning, conventional PLA has Tg ≈ 60 °C, which is too high for safe in vivo actuation [239]. Other strategies (copolymerizing with low-Tg segments, or using biodegradable polyurethanes) similarly position Ttrans in the 37–45 °C range. The permanent phase in thermally active SMPs can be either covalent crosslinks or crystallites that do not melt at body temperature. For example, semi-crystalline SMPs use the crystalline domains as permanent anchors (melting well above actuation), while amorphous thermosets use chemical crosslinks. In all cases, the fundamental mechanism is entropy-driven relaxation of polymer chains upon heating: when the temporary shape is heated above Ttrans, chain segments gain mobility and the internal stresses drive the network to revert to the stored permanent configuration [240]. In biomedical practice, thermally actuated SMPs have been proposed for self-expanding stents, “smart” sutures, and shape-changing scaffolds, taking advantage of safe heat sources (body heat or mild external warming) to trigger deployment without free catalysts or electrical currents.

### 4.2. Light-Activated SMPs

Light-activated SMPs exploit photothermal or photochemical effects to achieve shape recovery with remote optical control. In a common design, a photosensitive filler or chromophore is dispersed in the polymer so that illumination (often near-infrared, NIR) generates local heating. Because NIR penetrates tissue, SMPs with NIR-absorbing nanoparticles or dyes can be actuated transdermally. For example, incorporating polydopamine or gold nanorods into an SMP matrix enables near-IR light to rapidly heat the polymer above Tg and trigger recovery in seconds, while spatially confining the heating to illuminated regions [241]. Wang et al. demonstrated an epoxy-acrylate SMP with 0.1 wt% polydopamine nanoparticles that recovered from a bent state under 808 nm illumination (1 W/cm^2^) in about 60 s [242]. In general, light activation offers the advantages of remote, on-demand triggering and the ability to precisely focus or pattern the stimulus. Photochemical SMPs use UV or visible light to drive molecular switches (for example, azobenzene isomerization) embedded in the polymer, but in biomedical uses, UV is often avoided. Thus, most light-responsive biomedical SMPs rely on photothermal mechanisms: the light-absorbing agent (dye, nanotube, nanoparticle, or melanin-like polymer) converts NIR light to heat, heating the polymer matrix above its transition temperature without bulk heating of the surrounding tissue. This strategy can achieve fast actuation and reprogrammable shape changes with high spatial control, making it attractive for applications like photo-driven micro-actuators or on-demand scaffold reshaping [243].

### 4.3. pH-Sensitive SMPs

pH-sensitive SMPs use changes in acidity to switch between temporary and permanent shapes. In these systems, the switching phase contains ionizable or hydrolyzable groups whose state depends on pH. Two main mechanisms are employed: (1) ionizable functional groups (e.g., carboxyls, amines) that swell or change hydrogen-bonding under acidic/basic conditions, and (2) acid-labile covalent linkages in the polymer backbone (e.g., acetal, imine, hydrazone bonds) that cleave at low pH. Upon exposure to an acidic environment, protonation or bond hydrolysis causes polymer chains to loosen (e.g., H-bonds break, network degrades), allowing the SMP to switch back to its permanent shape. For example, a polyurethane SMP containing pendant carboxylic acid groups will collapse (due to protonation and hydrogen-bond disruption) when placed in an acid buffer, recovering its stored shape [244]. Tan et al. reviewed many biomedical polymers with acid-sensitive linkers: introducing hydrazone, acetal, or imine bonds into the network yields an SMP that is stable at neutral pH but rapidly relaxes shape in acidic (tumor-like or endosomal) conditions [245]. pH-activated SMP hydrogels have been demonstrated for drug delivery and tissue engineering: for instance, a dual-network hydrogel can be deformed and fixed at pH 7, then recover its shape in a pH~5 environment as an example of an acid-triggered SMP. The design principles for pH-SMPs include selecting functional groups with pKa’s around the target transition pH (often physiological or pathological pH ranges) and ensuring adequate crosslinking so that only under the trigger pH the network loosens [246]. Because different tissues and cellular compartments have characteristic pH (e.g., blood ~7.4 vs. tumor microenvironment ~6.5), pH-sensitive SMPs can be engineered to deploy specifically under those conditions, for example, as stents that expand in the acidic gastric environment or as shape-memory drug capsules that open in the intestinal pH.

## 5. Methods for Creating Polymer Scaffolds

An ideal bone scaffold must be biomimetic, biodegradable, and possess a porous structure that facilitates cell attachment, proliferation, and differentiation. Additionally, it must have sufficient mechanical strength to remain stable at the implantation site while minimizing immunogenic risk [247].

One simple method to fabricate such porous polymeric scaffolds is solvent casting. In this process, a chosen polymer is dissolved in an organic solvent, and a porogen such as sodium chloride (NaCl) is added to generate a polymer–porogen matrix. As the solvent evaporates, it leaves behind a solidified polymer structure with controlled porosity. While effective, this method is limited by its ability to control pore shape and interconnectivity [248,249].

Phase separation is another common technique used to create porous polymer scaffolds. This method utilizes thermal changes to drive the separation of a polymer such as poly(L-lactic acid) (PLLA) dissolved in two immiscible solvents. At lower temperatures, these polymer solutions become thermodynamically unstable. When heated, they become saturated, leading to a separation into a polymer-rich phase and a solvent-rich phase. Upon subsequent cooling, the polymer-rich phase solidifies into a high-porosity structure, while the solvent-rich phase is removed through extraction, sublimation, or evaporation [250].

Freeze-drying (lyophilization) is a versatile approach to producing porous polymer scaffolds without the use of porogens. In this technique, a water-based polymer solution is frozen, causing ice crystal formation. The polymer aggregates in the interstitial spaces between the ice crystals, and upon sublimation of the ice, a porous scaffold structure is obtained [251].

The directionality of freezing significantly impacts the morphology of the scaffold pores. Directed freezing refers to the alignment of ice crystals from a low to high temperature gradient, producing scaffolds with unidirectional, aligned pores. This method enables the fabrication of a wide range of porous structures using polymers in emulsions, solutions, or colloidal suspensions [252].

Emulsion-freezing is another scaffold fabrication technique that involves mixing polymer or ceramic materials dissolved in a solvent with water to form an emulsion. This emulsion is then cast into molds and frozen to induce phase separation. Subsequent freeze-drying removes the solvents and water, resulting in a porous structure [253].

Electrospinning is a highly adaptable and scalable technique for fabricating nanofibrous scaffolds that closely mimic the morphology of native extracellular matrix, making it ideal for tissue engineering applications. With a standard setup—comprising a high-voltage power supply, syringe pump, and various collector types—researchers can finely adjust fiber diameter and porosity by modulating solution properties (e.g., viscosity, conductivity), process parameters (e.g., voltage, flow rate), and ambient conditions (e.g., humidity, temperature) [254,255]. Electrospun scaffolds exhibit large surface-to-volume ratios and interconnected pore networks, promoting cell adhesion, proliferation, and controlled drug release, while advanced configurations like multi-fluid and multilayer electrospinning further enhance structural complexity and functionality [256].

**Figure 8:**
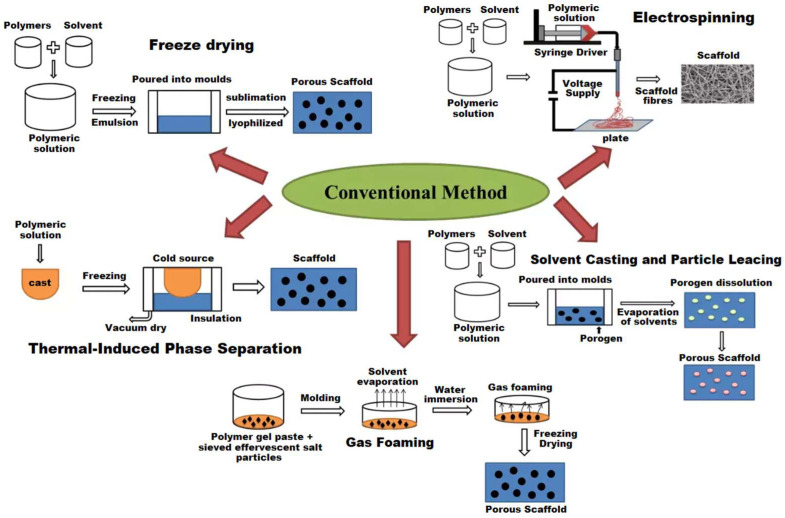
Diagrammatic illustration of traditional techniques used for scaffold fabrication. Recreated from [12]. (The figure is available under Open Access).

Visual representation of traditional techniques used for scaffold fabrication is provided in Figure 8.

Phase separation methods include nonsolvent-induced phase separation (NIPS) and thermally induced phase separation (TIPS). In NIPS, a polymer is dissolved in a solvent, cast, briefly exposed to air, and then immersed in a nonsolvent bath. Contact between the polymer solution and nonsolvent induces phase separation, resulting in polymer-rich and polymer-poor regions, with the solidified polymer-rich phase forming a porous structure [257,258].

TIPS involves preparing a homogeneous polymer solution at elevated temperatures, followed by cooling to initiate phase separation. Solidification of the polymer-rich phase forms the scaffold, while the solvent-rich phase is removed to create pores. TIPS can occur via solid–liquid (S-L) or liquid–liquid (L-L) mechanisms. In S-L separation, the solvent crystallizes upon cooling and is removed to leave pores. L-L separation results from phase coexistence at specific temperature and concentration conditions [259,260].

**Figure 9:**
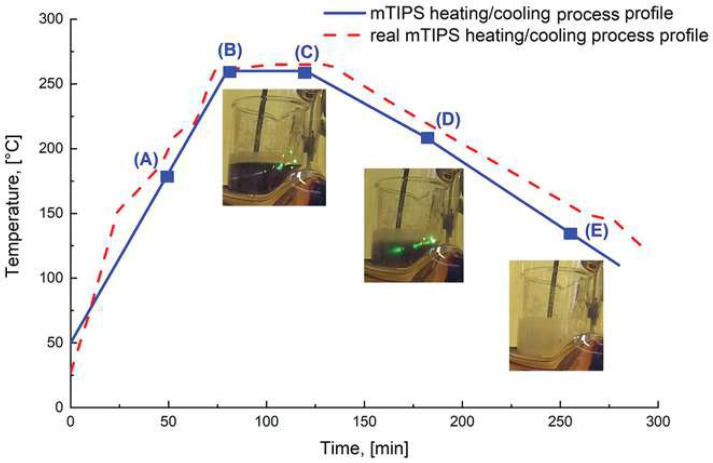
Diagrammatic representation of the TIPS method. The solid blue curve represents the target temperature profile for heating and cooling, while the red dashed curve indicates the actual temperature changes. Photographs inserted along the timeline show the state of the PEEK–4PPH mixture at key stages: (A) Melting of 4PPH and beginning of mixing; (B) temperature held steady with continuous stirring; (C) formation of a clear solution after complete polymer dissolution, followed by stirrer removal and start of cooling; (D) gelation phase observed in PEEK and PEKK systems as the polymer separates into a gel-like state; (E) crystallization of 4PPH, effectively solidifying the system [261]. (The figure is available under Open Access).

The TIPS method for fabricating porous high-performance polymers involved several controlled steps. In the study [261], a mixture of polymer and 4PPH powders was prepared and heated in a thermostatically controlled vessel until the 4PPH melted completely. The process is shown in Figure 9. Stirring was initiated to form a clear polymer solution, and the temperature was maintained for 30 min to ensure full dissolution. The mixture was then cooled gradually to 120 °C, followed by rapid cooling to room temperature to solidify the structure. Finally, the samples were purified via 48-h Soxhlet extraction in ethanol and dried at 60 °C for 24 h to achieve a stable weight.

Gas foaming involves introducing gas bubbles into a polymer matrix, often using foaming agents such as water (H_2_O), fluoroform, nitrogen (N_2_), or carbon dioxide (CO_2_). The polymer is compressed and saturated under pressure until bubble formation occurs [262,263].

Gas bubbles ranging from 100 to 500 µm can be generated using this approach [264,265]. The pore size is controlled by adjusting the polymer-to-foaming agent ratio. Gas formation may also occur via chemical reactions that release gases like N_2_, resulting in highly porous foam networks [266]. Though this method uses non-toxic solvents, it suffers from poor pore interconnectivity and non-porous external surfaces, limiting its utility [267].

Table 2 contains information on the advantages and limitations/drawbacks of every aforementioned method.

## 6. Available Biodegradable Devices

Interventional devices crafted from smart biodegradable polymers are advanced medical tools—like stents, sensors, drug-delivery implants, or wound dressings [275]—that combine two key features: (i) smart responsiveness [276]: they react to physiological triggers (e.g., temperature, pH, mechanical stress) to change shape, release drugs, self-heal, or conduct sensing; (ii) biodegradability [277]: after fulfilling their function, they safely break down within the body into non-toxic components, eliminating the need for surgical removal.

In this section, the discussion is primarily focused on biomedical devices that integrate both biodegradability and smart functionalities, such as shape memory, stimuli responsiveness, or self-healing behavior. Only those systems that exhibit at least one form of active responsiveness (e.g., to thermal, pH, enzymatic, electrical, or magnetic stimuli) while also being capable of in vivo degradation or resorption were included. This scope reflects the growing emphasis on transient biomedical systems designed to perform a specific function and then safely degrade without the need for surgical removal—an increasingly important requirement in fields such as minimally invasive surgery, tissue engineering, and controlled drug delivery. Non-biodegradable smart systems (e.g., permanent shape-memory alloys or silicone-based sensors) were excluded from the core analysis unless they directly informed or contrasted with biodegradable counterparts in terms of mechanism or performance. By narrowing the focus to dual-functional systems, this review aims to highlight the synergistic potential of smart and biodegradable materials in next-generation biomedical devices.

The Biostar device (NMT Medical, Boston, MA, USA) was the first partially bioresorbable device designed for percutaneous closure of atrial septal defects (ASDs) and patent foramen ovale (PFO) in humans [278,279].

The Double BioDisk (DBD) (Cook Medical, Bloomington, IN, USA) is another partially biodegradable occlusion device for ASD closure, building upon the Monodisk [280] and the single-disk BioDisk designed for PFO closure [281].

The Carag Bioresorbable Septal Occluder (CSBO) (CARAG AG, Baar, Switzerland) is a self-centering device composed of a PLGA bioresorbable frame and two opposing foldable polyester membranes [282].

The Pancy^®^ Occluder (Shanghai Mallow Medical Instrument Co., Ltd., Shanghai, China) is a partially biodegradable PFO occluder composed of a PDO dual-disk frame, a PET interlayer membrane, and degradable nylon thread [283].

In 2010, Duong-Hong D. and colleagues introduced a fully biodegradable septal defect occluder with a double-umbrella design, made of two self-expanding umbrella disks constructed from PCL and coated with PLC, along with eight symmetrically arranged spokes fabricated from poly(lactic-co-ε-caprolactone) (PLC) [284].

The Chinese Lantern (CL) occluder, developed by Venkatraman S.S.’s team in 2011, is a fully biodegradable PFO/ASD device, presenting a novel structural design [285].

Another innovation is the PCL-PLGA/collagen occluder, a biodegradable ASD device featuring a PCL skeleton created via microinjection molding and nanofibrous PLGA/collagen membranes produced through electrospinning [286].

In 2012, a fully biodegradable ASD occluder, modeled after the improved Amplatzer design, was produced and evaluated in animal studies [287].

The Absnow™ PLLA occluder (Lifetech Scientific, Shenzhen, China) is a fully bioabsorbable device for transcatheter ASD closure [288].

The Memosorb^®^ PFO occluder (Shanghai Shape Memory Alloy Co., Ltd., Shanghai, China) is another fully biodegradable device, developed from PLA-based ASD occluders [289].

Finally, the first-generation BAO device introduced by Shinoka T.’s team is fabricated from 4–0 poly(l-lactide-co-ε-caprolactone) (PLCL) and 15.2 µm biodegradable PGA polymers. This symmetric, double-disk structure is intended for ASD and PFO closure [290].

Table 3 provides detailed information on design, working principle, role of polymers, and advantages and disadvantages of every aforementioned device.

Traditional metallic alloy occluders for patent foramen ovale (PFO) closure are associated with certain complications and may restrict transseptal access to the left atrium for future interventions targeting left-sided heart conditions. This has led to increasing interest in novel biodegradable occluders (NBOs) as a more flexible and biocompatible alternative. The purpose of one study was to evaluate the role of transesophageal echocardiography (TEE) in both the diagnostic and anatomical assessment of PFO, as well as its postprocedural utility following transcatheter closure using an NBO [292].

Some resorbable scaffolds such as the Igaki-Tamai stent (Kyoto Medical Planning, Japan), the ABSORB scaffold (Abbott Cardiovascular, Plymouth, MN, USA), and the DEsolve platform (Elixir Medical Corporation, Milpitas, CA, USA) have already entered clinical use, with many others currently undergoing preclinical and clinical evaluation [293].

## 7. Discussion

The convergence of smart and biodegradable polymers represents a transformative leap in biomedical engineering, offering dynamic functionality, biocompatibility, and clinical versatility. One of the most compelling advances in this space is the development of shape memory polymers (SMPs), which enable devices to be deployed in minimally invasive configurations and later activated in situ using external stimuli such as heat, light, or pH change [30]. Compared to traditional shape memory alloys like Nitinol, SMPs offer greater flexibility, higher recoverable deformation (up to 800%), and the potential for remote activation [31,32]. These properties make SMPs particularly suited for applications such as self-tightening sutures and drug-eluting stents [33,34,35,36].

Multifunctional integration is a recurring theme across the smart polymer landscape. Incorporating antimicrobial agents like silver nanoparticles into PCL-based SMPs adds an essential infection-resistant dimension to devices like wound dressings and hemostatic agents [9,64]. Likewise, conductive additives such as carbon nanotubes enhance electrical responsiveness, supporting the use of SMPs in hemostasis and nerve regeneration [55,58,59,60,61].

In tissue engineering, the alignment of scaffold architecture with extracellular matrix (ECM) geometry is critical. Electrospinning and 3D printing are at the forefront of scaffold fabrication, enabling fine-tuned control over porosity, mechanical strength, and bioactive loading [11,12]. These scaffolds promote specific cell behaviors such as alignment and differentiation, especially when incorporating conductive or bioactive materials like polypyrrole or hydroxyapatite [16,62].

Importantly, the pairing of synthetic polymers (e.g., PLGA, PCL, PLA) with natural polymers or bioceramics yields composite scaffolds that benefit from both controlled degradation and enhanced bioactivity [14,15,16,17]. This hybrid strategy addresses key limitations of synthetic polymers, such as poor bioadhesion or acidic degradation byproducts, while improving osteoconductivity and tissue integration [50,53,89].

In the realm of drug delivery, smart polymers facilitate controlled, localized, and stimuli-responsive release, with hydrogel-based systems emerging as particularly effective. These systems can mimic physiological responses, altering permeability or swelling in response to temperature or pH changes to deliver therapeutic agents more efficiently [5,6,130,131]. Moreover, micellar systems and electrospun fibers provide tailored release kinetics suited to various medical needs, from transdermal patches to implantable drug reservoirs [81,88].

While the research shows promise, scalability, long-term biocompatibility, and regulatory hurdles remain challenges. Most fabrication methods, although effective in lab settings, face translation issues due to reproducibility, sterilization, and cost. Additionally, the immune system’s response to hybrid or synthetic materials needs deeper study to avoid fibrous encapsulation or chronic inflammation [54,55].

For vascular and endovascular devices, shape-memory polyurethanes and poly(lactide) derivatives are promising. For example, photo-activated polyurethane SMP foams have been demonstrated for self-expanding aneurysm stents [294]. For scaffold implants requiring high porosity and vascularization, polycaprolactone (PCL) is often chosen; PCL scaffolds can be seeded with cells and growth factors to rapidly induce blood-vessel formation [295]. For load-bearing bone devices, stiffer polyesters like PLLA or PLGA are preferred due to their higher modulus and slower degradation. As one group showed, an origami-inspired PLA scaffold can be highly compressible yet recover its shape, illustrating PLA’s utility in devices that must deform on deployment. For drug delivery systems, it is worth highlighting PLGA copolymers: PLGA is biocompatible, offers tunable degradation by adjusting the lactide:glycolide ratio, and has many FDA-approved drug formulations [296]. Each assignment is grounded in literature, e.g., PLGA is widely cited as the “gold standard” for controlled release due to its safety and versatility, while PCL’s ductility and compatibility make it ideal for soft-tissue scaffolds and vascular grafts.

In a related context, reference [297] introduces an innovative use of the Hartmann–Sprenger effect for natural gas pressure regulation through energy separation mechanisms. This study presents a quasi-isothermal, non-thermal pressure-reduction method utilizing nozzle–resonator assemblies to transform pressure energy into heat without requiring external energy sources. While not directly related to biodegradable polymers, the research highlights how strategic material and system design can contribute to energy efficiency and environmental sustainability—principles that are equally central to the advancement and implementation of biodegradable polymer technologies.

## 8. Future Directions and Outlook

The convergence of smart and biodegradable polymer technologies is poised to transform biomedical engineering, particularly in the fields of regenerative medicine, minimally invasive devices, and personalized therapeutics. While notable progress has been made, several avenues remain open for future research and innovation:

1. Multifunctionality Through Material Integration

Future materials will increasingly combine multiple functionalities—such as shape-memory, conductivity, antimicrobial activity, and controlled degradation—into a single platform. For example, shape-memory polymers (SMPs) with integrated photothermal or magnetothermal triggers could enable remote actuation without the need for invasive procedures. Similarly, incorporating bioactive or immunomodulatory agents directly into biodegradable scaffolds can support simultaneous tissue regeneration and inflammation control [298].

2. Dynamic and Stimuli-Responsive Systems

Smart polymers that respond to complex biological cues (e.g., enzymatic activity, oxidative stress, glucose levels) rather than only external stimuli like temperature or pH are a promising direction. These materials could deliver drugs or change mechanical properties in real-time based on the tissue microenvironment. For example, ROS- or MMP-responsive polymers are being investigated for applications in cancer therapy and wound healing [299].

3. Four-Dimensional Printing and Personalized Implants

Additive manufacturing (3D printing) will evolve into 4D printing, where smart polymers are printed into constructs that change shape or function over time in response to stimuli. This will enable patient-specific, self-deploying implants, stents, and tissue scaffolds with tailored mechanical and degradation properties. Biodegradable SMP inks already show promise in 4D-printed vascular occluders and sutures [300,301,302].

4. Bioinspired and Self-Healing Materials

Nature-inspired polymers—mimicking bone, cartilage, or skin—are being explored to match the nonlinear, time-dependent behavior of native tissues. Combining biodegradable backbones with self-healing capabilities (e.g., via hydrogen bonding, disulfide bridges, or Diels–Alder reactions) can extend device lifetime and reduce foreign body responses [303].

5. Improved Clinical Translation

Despite strong laboratory data, few smart biodegradable polymers have reached clinical use. Future efforts should emphasize long-term biocompatibility studies, standardization of degradation metrics, and cost-effective manufacturing. Regulatory strategies need to catch up with the dynamic behavior of these materials, which challenge existing medical device classification systems [303].

6. Integration with Electronics and Biosensors

There is growing interest in combining soft, degradable polymers with implantable biosensors and transient electronics for closed-loop therapeutic systems. This could enable real-time monitoring of healing, drug delivery feedback, or tissue stress, with the polymer scaffold gradually degrading after fulfilling its function [304,305,306].

## 9. Conclusions

Smart and biodegradable polymers are redefining the frontiers of regenerative medicine and interventional device design. Their capacity for environmental responsiveness, controlled degradation, and multifunctionality allows for unprecedented integration into clinical settings, particularly in scaffolds, occluders, and drug delivery systems. These materials promise minimally invasive, patient-specific solutions that align with the evolving landscape of precision medicine. As the field moves forward, the fusion of polymer chemistry, nanotechnology, and additive manufacturing will continue to drive innovations. However, challenges in clinical translation and large-scale production must be addressed to fully realize their transformative potential.

Out of all the discussed materials, PLGA (poly(lactic-co-glycolic acid)) currently remains the leading candidate for biodegradable drug delivery. PLGA is FDA-approved, biocompatible, and its degradation rate and mechanical properties can be finely tuned by the lactic/glycolic ratio. It has been called the “gold standard” of biodegradable polymers for controlled release, and numerous clinical formulations use PLGA-based microspheres and nanoparticles. Because its breakdown products are simply CO_2_ and H_2_O, it has a proven safety profile. In conclusion, PLGA’s track record (multiple approved drug-delivery products) and customizable behavior make it the most promising material for future smart drug delivery devices.

## Figures and Tables

**Table 1:** Comparison to other reviews on this topic.

Comparison Review (Title, Year, Journal)	Limitations of That Review	Strengths of the Current Review
Kurowiak et al., “Biodegradable Polymers in Biomedical Applications: A Review” (2023, IJMS) [22].	– Broad survey of natural vs. synthetic biodegradable polymers; general uses in TE, drug delivery, implants. – No specific focus on smart functions (e.g., stimuli-response) or advanced device designs. – Omits discussion of shape-memory or electrically conductive polymers.	– Explicitly covers smart functionalities, e.g., shape-memory polymers for minimally-invasive implants and vascular occluders. – Includes conductive polymers and composites (for drug release, antimicrobial). – Describes advanced fabrication methods (electrospinning, 3D printing) for porous scaffolds. – Highlights emerging trends (ionic doping, nanocarriers, personalized implants).
Socci et al., “Polymeric Materials, Advances and Applications in Tissue Engineering: A Review” (2023, Bioengineering) [23].	– Focuses on conventional polymer scaffolds and TE across eight tissue types (epithelial, bone, vascular, etc.). – Emphasizes composition and porosity but covers “smart” materials only briefly. – Lacks depth on stimuli-responsive behavior or device-specific design (e.g., no detailed section on polymeric occluders or shape-memory devices).	– Highlights shape-memory and self-healing polymers as smart devices (e.g., self-tightening sutures, shape-fitting stents). – Details conductive/composite biomaterials enabling drug delivery and antibacterial functions. – Emphasizes advanced scaffold fabrication (AM/3D printing for custom implants). – Treats drug-delivery and medical devices (e.g., occluders) as integral to TE, linking materials to specific clinical uses.
Khan et al., “Biodegradable Conducting Polymer-Based Composites for Biomedical Applications: A Review” (2024, Polymers) [24].	– Narrowly scoped on electrically conductive biodegradable polymers. Concentrates on conductivity vs. biodegradation trade-offs for implants and antimicrobial uses. – Does not address other smart polymers (like SMPs or stimuli-responsive hydrogels) or broad TE scaffolds. – Lacks coverage of fabrication methods beyond blending (no focus on AM or nanostructuring).	– Encompasses all smart polymer classes, not just conductive ones. Includes SMPs (shape-memory) and responsive hydrogels in addition to conductive composites. – Connects material properties to device function (e.g., how conductivity, degradability, porosity affect stem-cell scaffolds). – Reviews multiple fabrication techniques (electrospinning, 3D printing, freeze-casting) for customized scaffolds. – Discusses multifunctional applications (drug-eluting implants, antibacterial devices) in one framework.
Balcerak-Woźniak et al., “A Comprehensive Review of Stimuli-Responsive Smart Polymer Materials” (2024, Materials) [25].	– Surveys smart polymers by stimulus type (physical, chemical, biological) in a very broad way. – Covers classification (light-, pH-, thermo-responsive, etc.) across many fields (including electronics, agriculture). – Lacks focused coverage of biomedical implementations (only general examples, not device-specific). – Does not delve into bio-compatibility, degradability, or specific TE/device challenges.	– Applies smart polymer concepts directly to medical devices, e.g., smart scaffolds that guide stem-cell differentiation. – Emphasizes biodegradability as key (unlike generic smart materials reviews) and ties it to clinical benefits. – Includes examples of combined smart functions (e.g., SMPs with conductivity for drug delivery; hydrogels with mechanical/rheological control). – Details emerging biomedical techniques (3D printing of biodegradable devices, ionic doping to tune properties).
El-Husseiny et al., “Stimuli-responsive Hydrogels: Smart State-of-the-Art Platforms for Cardiac Tissue Engineering” (2023, Front. Bioeng. Biotechnol.) [26].	– Narrow focus on cardiac tissue engineering using smart hydrogels. Reviews types of hydrogels and their role for heart repair. – Excludes other polymers (elastomers, SMPs, etc.) and other tissues or devices. – Does not address manufacturing methods (beyond hydrogel chemistry) or interventional devices (occluders, stents).	– Goes beyond hydrogels to cover all smart/biodegradable polymers (SMPs, composites, nanofibers) for various tissues. – Connects materials to specific device examples (e.g., polymeric occluders, stents, sutures), not just matrix scaffolds. – Emphasizes fabrication innovation (e.g., 3D-printed personalized implants). – Integrates drug delivery (multifunctional nanocarriers in scaffolds) into the TE context, an aspect not in the hydrogel-only review.
Li et al., “Recent Development of Biodegradable Occlusion Devices for Intra-Atrial Shunts” (2024, Rev. Cardiovasc. Med.) [27].	– Ultra-focused on biodegradable ASD/PFO occluders (heart implants). Summarizes device designs, materials and biodegradation for septal defect closure. – Omits broader TE topics (e.g., vascular grafts, stents, tissue scaffolds) and other smart polymer categories. – Does not discuss general fabrication trends (just device-specific design issues).	– Includes occluder devices in a wider biomaterials’ framework (e.g., comparing SMP-based occluders vs. permanent ones). – Puts occluders in context of TE scaffolds and drug delivery (this review cites polymeric occluders alongside tissue scaffolds and carriers). – Covers fabrication advances (AM and nanotechnology) that benefit all implant types, including occluders. – Highlights multifunctionality (e.g., biodegradable scaffolds that also release drugs).
Sustainable Robots 4D Printing (2023, Adv. Sustainable Systems)—Soleimanzadeh et al. [28].	– Focuses on 3D/4D printing of biodegradable soft sensors and actuators. – Does not address shape-memory polymers, polymeric occluders/stents, or multipurpose scaffolds (e.g., combining antimicrobial function) that are covered by this review.	– Explicitly covers shape-memory polymers for minimally invasive devices and multifunctional polymer composites (e.g., conductive/antimicrobial scaffolds for drug release). – Discusses key fabrication techniques like electrospinning and 3D printing, providing a broader materials and devices perspective.
Bio-based stimuli-responsive materials for biomedical applications (2023, Materials Advances)—Ma et al. [29].	– Surveys bio-derived (mostly polysaccharide) stimuli-responsive polymers. – Does not emphasize synthetic SMPs, conductive composites, or multifunctional drug/antimicrobial scaffolds. – Focuses on molecular stimulus mechanisms rather than device integration.	– Bridges smart synthetic and natural polymers, explicitly covering shape-memory and conductive polymers in medical devices. – Highlights device-specific examples (e.g., occluders, stents) and fabrication methods (electrospinning, 3D printing).

**Table 2:** Advantages and limitations/drawbacks of various methods for polymer scaffolds creation.

Fabrication Method	Advantages	Limitations and Drawbacks
Solvent Casting and Particulate Leaching (SC/PL)	Simple, low-cost, tunable porosity (50–90%), controllable pore size (5–600 µm) [268,269].	Limited scaffold thickness (<3–4 mm), poor interconnectivity; use of toxic solvents that may leave residues and compromise biocompatibility; inconsistent reproducibility
Gas Foaming	Solvent-free porosity creation (~85%), suitable for hydrophilic/hydrophobic polymers [270].	Poor mechanical properties, non-uniform pores, often closed external surfaces, often poor pore interconnectivity. Long processing times: saturation and depressurization cycles may require days, which is impractical for rapid prototyping
Thermally Induced Phase Separation (TIPS)	High pore interconnectivity, uniform porosity, suitable for thermoplastics [271].	Complex, user-sensitive process; long freeze-drying time; limited macropore size (~100–200 µm); specialized equipment needed
Freeze-Drying (Lyophilization)	High porosity (>90%), homogenous porous network, preserves bioactive agents (no heating) [272].	Energy-intensive and costly, slow processing, often small and irregular pores
Electrospinning	Economical, simple, flexible, produces ECM-like nanofibers with controllable diameters [273].	Low throughput; frequent nozzle clogging; uses toxic solvents; weak mechanical strength; difficult to form true 3D structures and achieve uniform cell distribution
Additive Manufacturing (3D Printing: FDM, SLA, SLS, Bioprinting)	High architecture control; reproducible; custom geometries; room-temperature or cell-compatible printing [274].	Limited resolution for micro/nano-pores; restricted material choices; some techniques require heat or UV (potential cytotoxicity); high equipment cost

**Table 3:** Summary of design, working principle, polymer role, advantages and disadvantages for specific biodegradable devices.

Device (Manufacturer)	Design/Working Principle and Polymer Role	Advantages	Disadvantages
BioSTAR (NMT Medical, Boston, MA, USA) [279].	Self-expanding double-disc nitinol frame (MP35N alloy, non-degradable) covered by a biodegradable acellular porcine-derived type-I collagen membrane. After deployment, the collagen layer fuses to the septum and is gradually absorbed (90–95% by ~24 months), allowing native tissue ingrowth. (The device was also heparin-coated to reduce thrombosis.)	– Encourages host-tissue remodeling: collagen replaced by endothelium/connective tissue. – Heparin layer lowers early thrombogenicity. – Good short-term closure (≈96% at 6 mo).	– Late complications reported: wire fractures and local inflammation (led to device withdrawal). – Permanent metal frame may still cause long-term issues (erosion, arrhythmia, etc.) before full degradation of collagen.
Double BioDisk (Cook Medical, Bloomington, IN, USA) [280].	Two connected nitinol rings (double-disc) covered with a bioabsorbable porcine small intestinal submucosa (SIS) membrane. The self-expanding device centers itself in the defect and can be redeployed. The SIS (styrene-isoprene-styrene) polymer membrane acts as a temporary barrier and promotes tissue growth.	– Rapid defect closure in animal models; complete occlusion with full incorporation by 6–52 weeks. – Minimal long-term inflammation; no thrombus seen due to quick endothelialization.	– Only preclinical data reported so far. – Like BioSTAR, it retains a permanent nitinol frame (not fully absorbable); only the membrane is biodegradable.
Carag CSBO (CARAG AG, Baar, Switzerland) [282].	Self-centering double-disc occluder with a PLGA bioresorbable polymer frame and two polyester fabric covers. (PLGA = poly(lactic-co-glycolic acid) copolymer.) The frame degrades in vivo (begins ~6 mo, gone by ~18–24 mo). X-ray markers (platinum/Phynox) are incorporated for visibility.	– Complete endothelialization by 3 months; frame fully degrades by ~24 mo. – No permanent metal frame remains after resorption. – Early human trials show high closure rates (100% ASD closure at 24 mo).	– Some chronic inflammation around polyester seen histologically. – Small early clinical series: 12-month closure was 100% (ASD) but only 50% (PFO). – The need for non-resorbable filaments (PEEK holders) and metal markers means not entirely free of permanent material.
Pancy^®^ Occluder (Shanghai, China) [283].	Double-disc PDO (polydioxanone) frame with interleaving PET membrane and degradable nylon suture. (PDO is a bioabsorbable polymer.) The discs self-expand to seal the PFO. In animal tests, the PDO framework began dissolving at ~3 mo and was mostly gone by 6 mo.	– Rapid biodegradation of frame (disc structures absorbed by ~6 mo in dogs). – High acute success: multi-center China study reports 95–100% PFO closure at 12 mo. – Similar dual-disc design as Amplatzer but without metal (frame will eventually disappear).	– Thrombus formation noted on right disc in ~6.8% of cases at 3–6 mo (resolved with anticoagulation). – Long-term data limited; device is new (available since ~2019). – Like others, still contains PET and nylon (some non-degradable components).
Double-Umbrella Occluder [284].	Fully biodegradable double-umbrella design for PFO: two self-expanding umbrella-shaped discs of PCL (polycaprolactone) coated with a PLC (poly(L-lactide–co–ε-caprolactone)) film, plus eight symmetrical PLC spokes. A stretchable stem fixes the left disc on septum; right disc seals the defect.	– Achieved stable position in animal tests with no residual shunt. – Complete endothelialization seen by 1 month in swine. – Fully degradable (no metal): PCL/PLC degrade in ~months.	– Moderate thrombus formation and inflammatory response at 1 month in swine, indicating biocompatibility needs improvement. – Novel shape may not anchor as well as metal frames. – Reported results only in small animal study (swine, short-term).
Chinese Lantern (CL) [285].	Fully biodegradable device: “lantern” structure with soft polymer “head/waist/tail” films (blends of PLC/PCL) and a structural skeleton (wires) also of PLC/PCL blends. A pull-fold mechanism deploys the device; the waist length is adjustable to septal anatomy. Made radio-opaque by added (unspecified) radiopacifier.	– Successfully implanted in swine: devices were stable, endothelialized by 1 mo with no thrombi. – Fully polymeric (no metal) with novel foldable design.	– Original version lacked sufficient anchoring strength and septal coverage for larger defects. – Only tested in two animals; no further animal or human data yet. – Details on polymers blend and long-term degradation were not fully reported.
PCL-PLGA/Collagen Occluder [286].	Novel biodegradable ASD occluder: micro-injection-molded PCL scaffold (frame) with electrospun PLGA/collagen nanofiber membrane covering. Double-disc shape mimics Amplatzer device. PCL (semi-crystalline polymer) provides elasticity and shape memory, while PLGA/collagen film acts as a barrier and promotes cell adhesion.	– In vitro compression resistance comparable to Amplatzer device. – Superior sealing (less leakage) than Amplatzer in bench tests. – Nanofibrous PLGA/collagen promotes cell growth (good biocompatibility).	– Only tested in vitro (no in vivo/animal data yet). – PCL long-term degradation is slow (~years), and mechanical performance in vivo remains unproven. – Manufacturing complexity (microinjection + electrospinning) may complicate scaling.
Fully Biodegradable ASD Occluder (improved Amplatzer, 2012) [287]	Double-disc device (Amplatzer-like) with PDO monofilament frame (0.298 mm thick) and PLA (polylactic acid) membranes. Tantalum markers embedded for X-ray. Compressed for catheter delivery and self-expands on release. (PDO frame is elastic yet bioabsorbable.)	– High procedural success in canine model; by 12 wk the device was fully endothelialized and the PDO frame largely degraded by 24 wk. – Low complications reported in preclinical study.	– PLA membranes require ≥2 years to fully resorb, meaning residual polymer presence long-term. – Thick PDO filaments make upscaling to larger sizes difficult. – Moderate inflammatory response at 8 wk (resolved by 24 wk).
Absnow™ PLLA Occluder (Lifetech, China) [291].	Fully bioabsorbable double-disc device: 0.15 mm PLLA wire mesh skeleton bonded to PLLA membranes on both discs and waist. Novel locking/unlocking handle allows device shape control during deployment. Seven platinum-iridium markers for visibility. Available in 6–32 mm sizes.	– In swine, 100% endothelialization by 3 mo and near-complete degradation by 36 mo. – Animal studies show few inflammatory signs after full degradation. – In first-in-human (5 children) trial: good short-term closure and safety.	– Early inflammation: more local reaction than nitinol device within the first year (though resolved by 3 yr). – Human trial (3-yr follow-up) noted 3/5 patients with residual shunts (1 large, 2 moderate) (suboptimal efficacy). – Design modifications likely needed to improve occlusion.
Memosorb^®^ PFO Occluder (Shanghai, China) [289].	Evolved from an earlier PLA-based occluder. Current design is fully biodegradable double-disc: PDO monofilament framework with PLLA membranes. Delivered via novel sheath/pusher with internal cable; “waist” formed by pentagonal skeleton between discs (improves fit in complex septa).	– Preclinical (sheep) results: new PDO-PLLA design fully endothelialized by 6 mo; PDO frame largely degraded by 24 mo. – No thrombus or tissue necrosis observed; PDO offers good initial strength and faster resorption than PLLA. – Inspired by clinically successful Memosorb VSD occluder.	– Earlier PLA-only version degraded very slowly (mostly intact at 2 yr) with mild inflammation at 2 yr. – Memosorb PFO (new design) has limited public data; current human trials ongoing. – Like other fully polymeric devices, long-term outcomes in diverse patients remain to be seen.
BAO (Bioabsorbable ASD/PFO) [290].	First-generation biodegradable occluder: symmetric double-disc with 20 mm (left)/15 mm (right) discs and 5 mm waist, made of PLCL (poly(lactide–co–ε-caprolactone)) and 15.2 µm PGA fiber. A 0.9 mm nitinol spring gives X-ray visibility. After poor initial fit, 2nd-gen removed PGA, thickened PLCL fibers, added PLCL knit layer, and lengthened waist to 7 mm.	– Fully bioresorbable (1st-gen polymers gone by ~1 yr). – The 2nd-gen showed better septal conformity and was fully covered by endothelium at 1 yr. – Mild inflammation only; design changes improved performance.	– The 1st-gen had inadequate septal apposition (only 3/4 animals implanted). – The 2nd-gen polymer now degrades slower (due to thicker fibers). – Still early animal data; human use not yet reported.
